# Impact of exercise interventions on physical fitness in breast cancer patients and survivors: a systematic review

**DOI:** 10.1007/s12282-022-01347-z

**Published:** 2022-03-12

**Authors:** Salvatore Ficarra, Ewan Thomas, Antonino Bianco, Ambra Gentile, Petra Thaller, Fulvio Grassadonio, Sofia Papakonstantinou, Thorsten Schulz, Nils Olson, Alexandra Martin, Christian Wagner, Anna Nordström, Hande Hofmann

**Affiliations:** 1 Sport and Exercise Sciences Research Unit, Department of Psychology, Educational Science and Human Movement, University of Palermo, Via Giovanni Pascoli 6, 90144 Palermo, Italy; 2 OAC-Outdoor Against Cancer, Europe-Wide Outdoor Sport and Exercise Network for Cancer Prevention, Prinzregentenstrasse 97, 81677 Munich, Germany; 3 International Centre for the Promotion of Education and Development (CEIPES), Via Francesco Maria Alias 20, 90145 Palermo, Italy; 4 Creative Thinking Development (CreThiDev), Solonos 8 & Empedokleus, 19009 Ntrafi Rafinas, Attiki, Greece; 5 Department of Sport and Health Sciences, Institute of Preventive Pediatrics, Technical University of Munich, Georg-Brauchle-Ring 60/62, D-80992 Munich, Germany; 6 Department of Sport and Health Sciences, Technical University of Munich, Uptown Munich-Campus D Georg-Brauchle-Ring 60/62, D-80992 Munich, Germany; 7 Naturfreunde, Bundesorganisation, Viktoriagasse 6, 1150 Vienna, Austria; 8 Department of Public Health and Clinical Medicine, Section of Sustainable Health, Umeå University, Umeå, Sweden; 9 Umeå School of Sport Sciences, Umeå University, Umeå, Sweden

**Keywords:** Breast neoplasm, Physical activity, Strength, Fatigue, Quality of life

## Abstract

**Background:**

This systematic review aims to identify the effects of exercise interventions in patients with breast cancer (BCP) and survivors (BCS) on selected variables of physical fitness.

**Methods:**

A comprehensive literature search was conducted using Medline and Scopus. Randomized controlled trials with isolated exercise interventions in BCP and BCS women (< 5 years from therapy completion) were included. The risk of bias (RoB) assessment was conducted using the Cochrane RoB-2-tool. Variables regarding cardiorespiratory fitness (CRF), strength (ST), fatigue (F) and health-related quality of life (HRQoL) were discussed.

**Results:**

Of the 336 studies initially identified, 22 met all the inclusion criteria and were deemed eligible. RoB assessment indicated that the studies had predominantly “some concerns” or had “low RoB”, with only 3 studies presenting a “high RoB”. The mean duration and frequency of exercise interventions were 19 weeks and 3 sessions/week, performed at moderate intensity (65% *V*O_2_max and 66% 1RM, for aerobic and resistance-training interventions, respectively).

**Conclusions:**

Exercise interventions seem to be a valuable strategy in BCP to avoid the decline of CRF, ST, F and HRQoL. Conversely, improved physical function among BCS is observed for the same variables. Resistance training and combined interventions seem to provide the most encouraging variations of the selected outcomes.

**PROSPERO registration ID:**

CRD42021237917.

**Supplementary Information:**

The online version contains supplementary material available at 10.1007/s12282-022-01347-z.

## Introduction

According to the World Health Organization, cancer is a leading cause of premature death (age < 70 years) in different countries [1]. Female breast cancer (BC) is the most frequently diagnosed cancer, nearing 2.3 million diagnosed cases in 2020 and ranking first for incidence in 159 out of 185 countries [2]. Its incidence is greater in high-and very high human development index countries, which likely occurs due to the increased prevalence of risk factors (e.g. estrogen replacement, contraceptive pills, high-fat, low-fiber diets, alcohol consumption, obesity, late pregnancy or non-pregnancy) as well as from increased early detection/diagnosis through mammographic screening [2, 3].

Currently, BC management and treatment combine different approaches in response to disease type, stage and progression and include targeted therapies, hormonal treatment, radiation and chemotherapy, and surgery [3]. Independent of the surgical technique (lumpectomy, mastectomy with or without breast reconstruction), surgery may require a substantial amount of time before recovering Health-Related Quality of Life (HRQoL) and can cause physiological and psychological side effects [3, 4]. Chemotherapy and radiation may also cause physical alterations and physiological side effects [5], which frequently include fatigue, insomnia, nausea, emesis, neuropathy, cardiotoxicity and strength impairment [5–7]. Fatigue (F), in particular, is the most frequently experienced side effect among all cancer typologies [8–10]. The frequent administration of additional standard BC therapies before (neoadjuvant therapies) and/or after surgery (adjuvant therapies, that occur when primary disease is defeated) can further cause additional side effects to what is already experienced by primary treatment [3, 11].

Still, despite the onset of new symptoms, neo- and adjuvant therapies have increased the survival rate among BC patients (P) [12, 13]. However, with the resultant increased life expectancy, side effects from therapy can also persist long term, which can further impact HRQoL [8, 9]. Consequently, detrimental effects on indices of physical fitness, including cardiorespiratory fitness (CRF) and strength (ST), are frequently observed [7]. CRF is related to cardiovascular health that is usually impaired in BCP and BCS, leading to premature mortality. ST attenuations can reduce functionalities and strongly limit independence, and therefore, HRQoL [14]. Within this context, BCP and BC survivors (S), could enter a vicious cycle wherein the symptoms and side effects of cancer and its treatment impair CRF and ST, increasing sedentary level. This is further exacerbated by rest prescriptions, which often occur because medical procedures do not always consider exercise for cancer patients [8, 15, 16]. Thus, to improve or maintain CRF through exercise, reducing rest, must be essential to lower the mortality derived by cardiovascular diseases (relevant cause of death for BCS) [17, 18]; while ST improvements are required to allow survivors and patients to maintain independency avoiding HRQoL declines.

However, due to BC complexity and its multiple side effects (both physiological and psychological), a single complementary approach is usually inadequate. Psychological support, physical therapy, acupuncture, massages, and behavioral changes management are commonly employed and administered with or without exercise therapy [19]. Among these complementary approaches, exercise is particularly effective in either preventing BC or mitigating decrements in indices of physical fitness in both BCP and BCS [20–27]. The current American College of Sports Medicine guidelines, suggest at least three times/week (30 min per session) of moderate aerobic training and an additional 2 sessions/week of resistance training (8–15 repetitions at 60% of 1-Repetition Maximum—1RM) for all cancer survivors [28]. The Exercise and Sports Science Australia position statement for cancer patients suggests moderate to high intensity exercise with a flexible multimodal approach, individualized to patients characteristics (therapy cycles, surgery, side effects) [29].

A previous systematic review and meta-analysis was conducted in 2006 that showed the beneficial effects of exercise interventions on several parameters of physical functioning and quality of life [20]. However, as scientific research has significantly advanced during the last 20 years, both in terms of methodological advancement and the quantity of research available, an update assessing the effects of exercise in BCP and BCS is warranted. Therefore, the aim of this systematic review is to determine the effects of isolated exercise interventions on selected variables of physical fitness and major symptoms in BCP and BCS, from published research in the last 20 years.

## Materials and methods

The review adhered to the guidelines of the Preferred Reporting Items for Systematic Reviews and Meta-Analyses [30] Statement. The current review protocol was registered in the PROSPERO database [Reg. ID CRD42021237917].

### Search strategy

The search strategy for this systematic review was conducted for peer-reviewed articles published between January 2000 and November 2020 using two databases: PubMed (MEDLINE) and Scopus. Original studies examining the effects of exercise interventions in BCP and BCS were screened. Preliminary research using “breast cancer” AND “exercise” to define the keywords through a snowball sampling was applied. The following keywords were identified and applied in the final search strategy: “breast cancer”, “breast neoplasm”; “exercise” and “physical activity”. An example of the full electronic database search for one database with the additional applied filters is provided in the Electronic supplementary material.

Reference lists were also screened in relevant studies and only available full texts were included. Authors were contacted to obtain the unavailable full text and studies were excluded when authors were unreachable or if they could not provide the full text.

An Excel (Microsoft Corp., Redmond, WA) spreadsheet was used to manage all potentially eligible study titles. After the title screening, duplicates were removed and eligibility criteria were then applied during the subsequent abstract and full-text screening. The selection process was conducted independently by two authors for title, abstract and full-text screening. A third author was consulted to resolve any disagreements between the two authors. The final searches were then implemented using the appropriate specifications of each database using the PICOS format (see Electronic supplementary material).

### Eligibility criteria

After the title screening, the following inclusion and exclusion criteria were applied during the abstract screening and in all full-text sections.

#### Patients

Studies that included female BCP currently in therapy or BCS that were < 5 years from the conclusion of all therapy (excluding hormonal therapy as it is usually administered as a long-term adjuvant therapy [31]) were selected. Both neoadjuvant and adjuvant therapy patients were considered as BCP. Due to the BCP/BCS characteristics, age and comorbidities were not considered as exclusion criteria. Male patients or the presence of other cancer diagnoses were excluded from analysis.

#### Intervention

Only exercise interventions ≥ 4 weeks were included. Studies with clear exercise protocols without any additional approach were included and studies that implemented physical therapy, psychological approach, mind–body therapy, nutritional advice or diet management were excluded. Since stretching has cardiovascular outcomes [32], studies with combined stretching interventions (e.g. during warm-up and cool-down) were excluded. Furthermore, studies with non-standardized protocols (e.g. personal training and/or individualized interventions) were excluded.

#### Comparators

We considered the groups within the studies which provided usual care and associated control groups (without any interventions). Others exercise interventions different from those deemed eligible within the “intervention” section were also considered comparators. Groups within included studies which provided interventions different from exercise or physical activity (e.g. relaxation, mind–body therapy) were excluded.

#### Outcomes

Four variables were included in this review: (1) cardiorespiratory fitness considered as an indicator of the cardiovascular and respiratory system capacity to deliver oxygen to tissues during activities [33]; (2) strength defined as the force generated by a specific muscle or muscle group [34]; (3) fatigue symptoms identified as a tiredness condition from which it is impossible to recover with rest [8–10]; and (4) Health-Related Quality of Life which represent the health status perception of the individual [35]. Only studies with pre- and post-intervention data on CRF, ST, F and HRQoL available were included in the review. Objectively measured data were included.

#### Study design

Only randomized controlled trials (RCTs) with BCP and BCS were included.

### Study record

Studies were categorized into two groups according to the type of population: (1) BCP and (2) BCS. Four subgroups for exercise intervention typology were subsequently created: (1) aerobic (A), (2) resistance training (RT), (3) combined aerobic and resistance training (COMB) and (4) Pilates and Yoga interventions. Pre- and post-intervention measures were extracted from tables, text, and graphs of each study. For graphical interpretation, data were extracted using WebPlotDigitizer (version 4.2, San Francisco, CA) software. All data were managed using tables created in Word (Microsoft Corp., Redmond, WA) and descriptive statistics were implemented with Excel (Microsoft Corp., Redmond, WA). Study characteristics, including the mean age of participants and the type (e.g., RT, COMB), length (weeks), frequency (sessions/week), and intensity of the intervention were reported as well as which outcomes were measured and the methods by which they were measured.

### Risk of bias assessment

Risk of bias (RoB) assessment was implemented through the Cochrane RoB 2 tool for RCTs, following the Cochrane Handbook for Systematic Reviews of Interventions [36]. The tool has five different domains used to generate the overall RoB. The RoB judgement for the second domain (RoB due to deviations from the intended interventions) was carried on to quantify the effect of assignment to intervention. Each domain was evaluated with one of the following options: “Low RoB”, “Some Concerns” and “High RoB”. Following the individual domain assessment, we then categorized studies with just 1 out of 5 risk domains with a “Some Concerns” judgement as a “Low RoB”. Studies with 2 or more “Some Concerns” judgements were judged as “Some Concerns”. Studies with 1 domain in “High RoB” were judged as “High RoB”. RoB for each study was evaluated by two authors and disagreements were resolved by negotiation. Only already available additional documents concerning protocol and/or statistical analysis plan were screened to assess RoB.

### Data processing

Results are expressed as means ± SD. Differences from baseline and post-intervention were obtained and reported in tables. Percentage differences between pre- and post-intervention were calculated. When only pre-test and variations were reported, post-test data were calculated. CGs (Control Groups) results were also extracted, but only percentage differences between pre- and post-test are reported. An overall mean percentage difference was obtained for every outcome included for both IGs (Intervention Groups) and CGs.

## Results

**Fig. 1:**
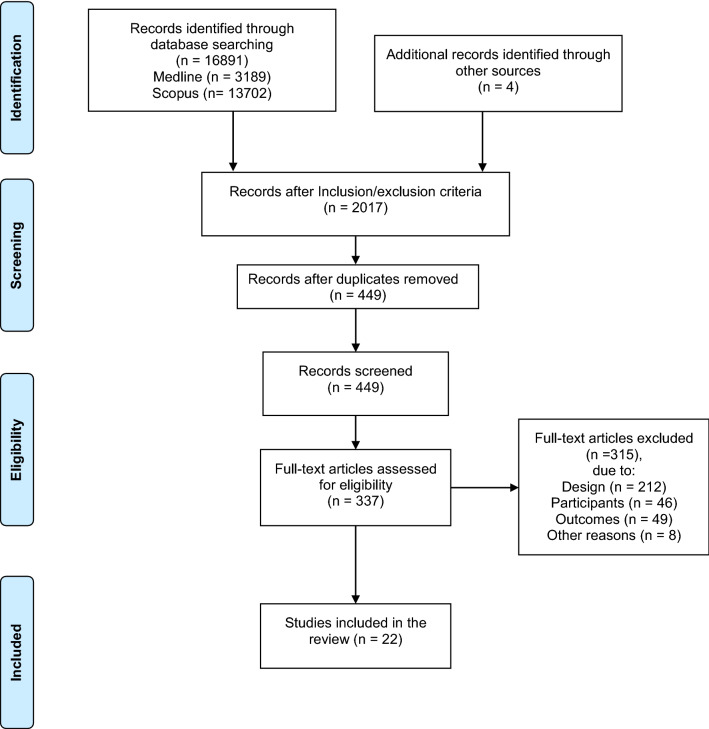
Flow diagram of the screening process. From Moher et al. [30]

After the initial search, 16,891 studies were identified. Four additional records were found through other sources. Following the title screening, 2017 relevant studies were detected, 1568 duplicates were removed and 449 remaining studies were deemed eligible. After the abstract screening, 112 records were excluded with reasons and 337 studies remained. After full-text screening, 22 studies were included in the review (Fig. 1). Characteristics of the studies included in the analysis are presented in Table 1.

**Table 1:** Study characteristics

Author, year	Sample size (n)	Mean age (y)	Intervention typology	Weeks (n)	Frequency (n/w)	Intensity (%)	Outcomes	Assessment tools
In therapy (ADJ)								
Cešeiko et al. 2019	27	RT 48.2 ± 6.7 CG 49.0 ± 8.0	RT^a^	12	2	85–90% 1RM	ST, F, HRQoL	Leg press 1RM test; EORTC QLQ-C30/BR23
Cešeiko et al. 2020	27	49.0 ± 7.0	RT^a^	12	2	85–90% 1RM	CRF	6 MWT
Courneya et al. 2013	298	50.0 ± 8.9	A–STAN^a^ A–HIGH^a^ COMB^c^	16	3	A: 55–75% of *V*O_2_peak RT: 60%-75% 1RM	CRF, ST, F, HRQoL	Maximal incremental exercise protocol; 10RM test; FACIT-F; SF-36
Schmidt et al. 2015	49	52.2 ± 9.9	RT^a^	12	2	60–80% 1RM	F, HRQoL	FAQ; EORTC QLQ-C30 / BR-23
Schmidt T. et al. 2015	67	RT 53.0 ± 2.6 A 56.0 ± 0.2 CG 54.0 ± 1.2	A^a^–RT^a^	12	2	RT: 50% h1RM	F, HRQoL	MFI-20; EORTC QLQ C-30/BR-23
Schwartz et al. 2007	66	A 48.3 ± 12.6 RT 50.1 ± 8.7 CG 46.3 ± 9.8	A^b^–RT^b^	24	4	n/a	CRF, ST	12MWT; Leg extension 1RM test
Segal et al. 2001	123	A 51.4 ± 8.7 A 51.0 ± 8.7 CG 50.3 ± 8.7	A^c^–A^b^	26	5	50–60% VO_2_max	CRF, HRQoL	mCAFT; SF-36
Steindorf et al. 2014	77	55.2 ± 9.5	RT^a^	12	2	60%–80% 1RM	F, HRQoL	FAQ; EORTC QLQ C-30/BR-23
**Tot**/*mean*	**734**	*50.9 ± 7.4*	–	*16*	*3*	*A 60% V*O_*2*_*max*—*RT 72%1RM*	–	–
Survivors								
Campbell et al. 2018	19	52.4 ± 6.2	A^c^	24	4	60%-80% HRR	CRF, F	Graded maximal treadmill exercise test; FACT-F
Courneya et al. 2003	52	59.0 ± 6.0	A^a^	15	3	70–75% VO_2_max	CRF, F, HRQoL	Incremental exercise protocol on a cycle ergometer; FACIT-F; FACT-G/B
Dieli-Conwright et al. 2018	91	53.5 ± 10.4	COMB^a^	16	3	RT: 60% and 80% 1-RM for upper and lower extremity, respectively A: 65–80% HRmax	CRF, ST, F, HRQoL	Single-stage submaximal treadmill test; 10RM test; BFI; FACT-G/B; SF-36
Hagstrom et al. 2016	39	51.9 ± 8.8	RT^a^	16	3	n/a	ST, F, HRQoL	Leg press 1RM test; FACIT-F; FACT-G
Kiecolt-Glaser et al. 2014	186	51.6 ± 9.2	Yoga^c^	12	2	n/a	F	MFSI-SF
Murtezani et al. 2014	62	52.0 ± 11.0	A^a^	10	3	50–75% HRR	CRF, HRQoL	12MWT; FACT-G/B
Nikander R. et al. 2007	28	A 52.5 ± 6.4 CG 51.3 ± 7.3	A^c^	12	3–4	n/a	CRF, ST	2-km walk test; Isometric Leg Extension
Nikander et al. 2012	67	COMB 53.7 ± 6.8 CG 52.6 ± 7.1	COMB^c^	48	3–4	n/a	CRF, ST	2-km walk test; Isometric Leg Extension
Northey et al. 2019	17	62.9 ± 7.8	A—HIIT^a^ A—MOD^a^	12	3	A(Moderate): 55–65% Peak Power A(HIIT): 105% Peak Power (90%HRmax) and self-selected active recovery	CRF	Maximal cycle ergometer incremental test
Odynets et al. 2019	70	Pilates 59.4 ± 1.2 Yoga 59.1 ± 1.4	Pilates^a^—Yoga^a^	48	3	Pilates: 45%-60% HRR	HRQoL	FACT-B
Saarto et al. 2012	500	A 52.3 (36–68) CG 52.4 (35–68)	A^c^	48	3–4	n/a	F, HRQoL	FACIT-F; EORTC QLQ C-30 / BR-23
Schmidt T. et al. 2012	15	58.0 ± 8.4	RT^a^	24	1	> 50% h1RM	F, HRQoL	EORTC QLQ-C30 / BR-23
Scott et al. 2020	117	LET 59.0 ± 9.0 NLET 58.0 ± 9.0	A—LET^a^ A—NLET^a^	16	3–4	70% *V*O_2_peak A(linear) 55%—> 95% *V*O_2_peak A(nonlinear)	CRF, F, HRQoL	Symptom-limited CPET; FACIT-F; FACT-G/B
Stan et al. 2016	16	63.0 ± 9.3	RT^b^	12	3–5	n/a	F, HRQoL	MFSI-SF; FACT-G/B
**Tot**/*mean*	**1279**	*55.7 ± 7.0*	–	*22*	*3*	*A 69%VO*_2_max/*HRR—RT 60%1RM*	–	–

*ADJ* adjuvant therapy, *CG* control group, *A* aerobic training, *RT* resistance training, *COMB* combined aerobic and resistance training, *STAN* experimental group that follow the Physical Activity Guidelines for Americans endorsed for cancer survivors by the American College of Sports Medicine and the American Cancer Society (75 min/week of vigorous aerobic exercise on 3 day/week), *HIGH* experimental group that follow double the STAN protocol (150 min/week of vigorous aerobic exercise on 3 day/week), *HIIT* high-intensity interval training, *MOD* Moderate intensity continuous aerobic exercise, *LET* linear intensity exercise training, *NLET* nonlinear intensity exercise training, *1/10RM/h1RM* one/ten repetition/s maximum/hypothetical one repetition maximum, *VO*_*2*_*peak/max* peak of oxygen consumption/maximal oxygen consumption, *RPE* rate of perceived exertion (based on Borg Scale), *HRR* heart rate reserve, *HRmax* maximal heart rate, *CRF* cardiorespiratory fitness, *ST* strength, *F* fatigue, *QoL* Quality of Life, *6/12MWT* 6/12 Minutes Walking Test, *mCAFT* modified Canadian Aerobic Fitness Test, *CPET* Cardiopulmonary Exercise Test, *FACIT-F* Functional Assessment of Chronic Illness Therapy—Fatigue, *FAQ* Fatigue Assessment Questionnaire, *MFI-20* Multidimensional Fatigue Inventory with 20 questions, *MFSI-SF* Multidimensional Fatigue Syndrome Inventory-Short Form, *BFI* Brief Fatigue Inventory, *EORTC QLQ-C30/BR23,* European Organization for Research and Treatment of Cancer Quality of Life Questionnaire-C30/BR23 Modules, *SF-36* Short Form Health Survey with 36 items, *FACT-G/B* Functional Assessment of Cancer Treatment—General/Breast

^a^Supervised intervention

^b^Unsupervised intervention

^c^Supervised and unsupervised intervention

A total sample of 2013 (734 BCP and 1279 BCS) participants were included in the quantitative analysis. The mean ages for BCP and BCS were 50.9 ± 7.41 and 55.7 ± 7.04 years, respectively. Studies were stratified according to BC status, with 8 studies performed in BCP [37–44] and 14 studies in BCS [45–58].

Adherence and/or attendance rate to training sessions was reported in 18/22 included studies. Studies among BCP (6/8) showed high-to very high adherence rate (79.9%) while only two reported attendance below 75% (71% and 71.5%) [40, 43]. Is not possible to determine whether adherence and/or attendance in BCP were related to intervention typology due to the limited number of studies.

Among BCS, high rates of adherence were also observed (83.6%), with 76% and 75.4% as minimum values in only two studies [49, 52] and only one study showing 62% attendance rate [55]. No differences regarding adherence were observed across intervention typology, weekly frequency and exercise intensity in both populations (BCP and BCS). However, higher adherence rates were observed for both groups in fully supervised [37–41, 44, 46–48, 50, 53, 54, 56, 57] vs. partially supervised/unsupervised interventions [39, 42, 43, 45, 49, 51, 52, 55, 58] (83.3% vs 71.8% mean BCP adherence; 85.5% vs 79.2% mean BCS adherence).

Overall attrition rate was reported in 19/22 studies. Mean attrition rate was 8.4%, and 11.8% for studies including BCP and BCS, respectively. The majority reported an overall attrition rate below 10%. Only two studies reported attrition rate above 20% both including BCS [56, 58].

Levels of evidence and the grades of recommendation are presented in the Electronic supplementary material.

### Risk of bias

**Fig. 2:**
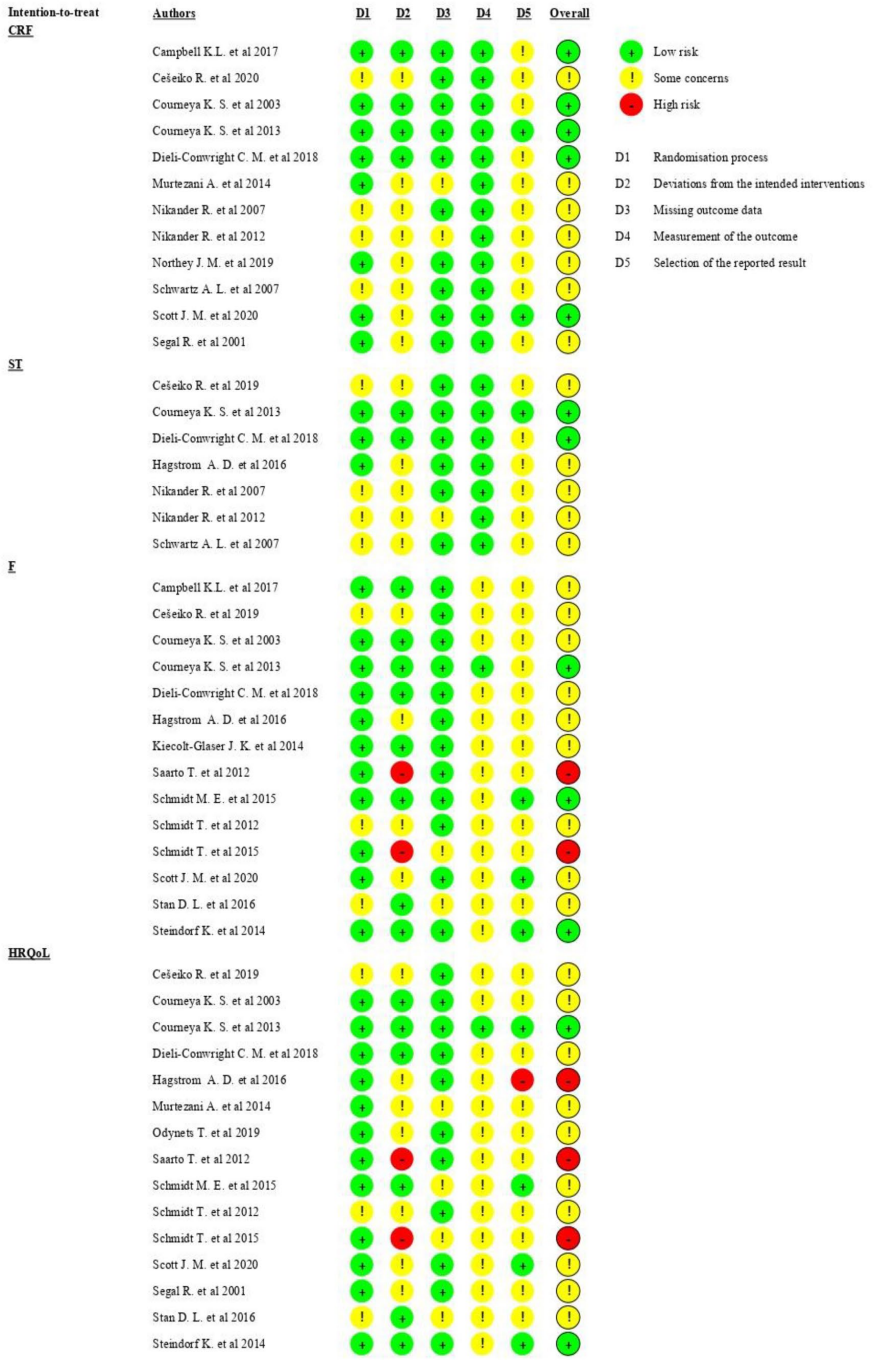
Risk of bias assessment summary, stratified by outcome. *D* domain, *CRF* cardiorespiratory fitness, *ST* strength, *F* fatigue, *HRQoL* health-related quality of life

RoB evaluations for each outcome are summarized in Fig. 2. Overall, the judgements predominantly exhibited “some concerns” mostly due to the lack of availability of additional documents. The variables F and HRQoL exhibited a higher RoB than CRF and ST due to the self-reported nature of the outcomes and a lack of additional documentation. The designation of “high RoB” was assigned only to three studies [41, 48, 55].

### In therapy

Table 2 presents results regarding the included studies with BCP and a specific training effects summary for each variable, as well as the comparisons with CGs pre-post percentage differences. There were 8 included studies conducted in BCP, with all of them in adjuvant therapy [37–44], comprising a total sample of 734 participants. Mean duration and frequency of interventions were 16 (range 12–26) weeks and 3 (range 2–5) sessions/week. The reported mean exercise intensity was 60% maximal oxygen consumption (*V*O_2_max) and 72% 1RM, for A and RT interventions, respectively. There were 4 studies with A interventions [39, 41–43], 6 studies with RT interventions [37, 38, 40–42, 44], and 2 studies that presented both A and RT experimental intervention groups [41, 42]. One study implemented a COMB intervention [39]. To be noted that, in the following lines and in Tables 2 and 3, regarding CRF, ST and QoL improved outcomes are represented by a percentage increase (+), while F symptoms represent improvements through a percentage decrease (−).

**Table 2:** Studies with in therapy BCP results pre–post-intervention and percentage differences within groups (both intervention and control group)

Author year	Type of exercise	Pre-value	Post-value	%Diff IG	%Diff CG
CRF					
Courneya et al. 2013—STAN	A	29.0 ± 6.4 ml/kg/min	25.6 ml/kg/min	− 11.7	/
Courneya et al. 2013—HIGH	A	28.9 ± 6.4 ml/kg/min	26.4 ml/kg/min	− 8.7	/
Schwartz et al. 2007	A	983.0 ± 289.0 m	1228.0 ± 322.0 m	+ 24.9	− 8.8
Segal et al. 2001—HB	A	25.9 ± 5.2 ml/kg/min	26.8 ml/kg/min	+ 3.5	0
Segal et al. 2001—S	A	25.5 ± 5.4 ml/kg/min	26.1 ml/kg/min	+ 2.4	/
Cešeiko et al. 2020	RT	491.4 m	538.0 m	+ 9.5	− 4.9
Schwartz et al. 2007	RT	1020.0 ± 357.0 m	1055.0 ± 177.0 m	+ 3.4	− 8.8
Courneya et al. 2013	COMB	27.5 ± 6.4 ml/kg/min	23.9 ml/kg/min	− 13.1	/
**Total**	**A**			** + 2.1**	− **4.4**
	**RT**			** + 6.4**	− **6.9**
	**COMB**			− **13.1**	**/**
ST					
Courneya et al. 2013 –STAN	A	83.7 ± 24.2 kg	86.2 kg	+ 3.0	/
Courneya et al. 2013 –HIGH	A	77.6 ± 24.7 kg	80.1 kg	+ 3.2	/
Schwartz et al. 2007	A	64.0 ± 26.0 kg	78.6 ± 30.5 kg	+ 22.8	+ 7.0
Cešeiko et al. 2019	RT	106.8 ± 22.8 kg	127.2 ± 26.4 kg	+ 19.1	− 9.1
Schwartz et al. 2007	RT	60.4 ± 31.8 kg	75.3 ± 34.5 kg	+ 24.7	+ 7.0
Courneya et al. 2013	COMB	87.1 ± 27.4 kg	95.7 kg	+ 9.9	/
**Total**	**A**			** + 9.7**	** + 7.0**
	**RT**			** + 21.9**	**– 1.1**
	**COMB**			** + 9.9**	**/**
F					
Courneya et al. 2013 –STAN	A	40.4 ± 9.3	34.2	+ 15.3	/
Courneya et al. 2013 –HIGH	A	40.6 ± 9.4	36.0	+ 11.3	/
Schmidt et al. 2015 -EORTC QLQ C-30	A	31.1 ± 26.4	48.0 ± 21.8	+ 54.3	+ 42.4
Schmidt et al. 2015 -MFI-20	A	8.8 ± 4.31	12.4 ± 4.4	+ 40.9	+ 29.8
Cešeiko et al. 2019	RT	33.5 ± 17.1	25.5 ± 15.5	− 23.9	+ 24.7
Schmidt et al. 2015	RT	36.4 ± 19.2	36.1 ± 20.6	− 0.8	/
Schmidt et al. 2015 -EORTC QLQ C-30	RT	22.2 ± 21.9	38.6 ± 17.4	+ 73.8	+ 42.4
Schmidt et al. 2015 -MFI-20	RT	9.3 ± 3.1	10.6 ± 3.2	+ 14.0	+ 29.8
Steindorf et al. 2014 -EORTC QLQ C-30	RT	42.0 ± 25.0	34.0 ± 28.0	− 19.0	/
Steindorf et al. 2014 – FAQ	RT	5.9 ± 2.2	5.4 ± 2.3	− 8.5	/
Courneya et al. 2013	COMB	40.7 ± 10.2	34.9	+ 14.3	/
**Total**	**A**			** + 17.1**	** + 36.1**
	**RT**			** + 5.9**	** + 32.3**
	**COMB**			** + 14.3**	**/**
HRQoL					
Courneya et al. 2013 –STAN	A	46.9 ± 7.4	44.0	− 6.2	/
Courneya et al. 2013 –HIGH	A	48.2 ± 8.1	45.7	− 5.2	/
Schmidt et al. 2015	A	30.4 ± 18.2	36.8 ± 18.0	+ 21.1	− 0.6
Segal et al. 2001 –HB	A	76.1 ± 15.6	81.8	+ 7.5	− 4.9
Segal et al. 2001 –S	A	76.5 ± 19.2	78.7	+ 2.9	/
Cešeiko et al. 2019	RT	67.2 ± 15.6	76.2 ± 14.3	+ 13.4	− 4.2
Schmidt et al. 2015	RT	61.5 ± 17.5	61.7 ± 18.3	+ 0.3	/
Schmidt et al. 2015	RT	24.8 ± 14.0	31.3 ± 15.9	+ 26.2	− 0.6
Steindorf et al. 2014	RT	59.0 ± 21.0	64.0 ± 25.0	+ 8.5	/
Courneya et al. 2013	COMB	47.9 ± 7.8	44.4	− 7.3	/
**Total**	**A**			** + 4.0**	− **2.8**
	**RT**			** + 12.1**	− **2.4**
	**COMB**			− **7.3**	**/**

Bold indicates percentage difference mean values for specific exercise intervention typology

+  increase*, − decrease*, * to be noted that regarding CRF, *ST and QoL* improved outcomes are represented by increases, while F symptoms represent improvements through decrease. *%Diff* percentage differences within group, *CRF* cardiorespiratory fitness, *ST* strength, *F* fatigue, *HRQoL* health-related quality of life, *A* aerobic training, *RT* resistance training, *COMB* combined aerobic and resistance training, *STAN* experimental group that follow the Physical Activity Guidelines for Americans endorsed for cancer survivors by the American College of Sports Medicine and the American Cancer Society (75 min/week of vigorous aerobic exercise on 3 day/week), *HIGH* experimental group that follow double the STAN protocol (150 min/week of vigorous aerobic exercise on 3 day/week), *HB* Home-Based Exercise Group, *S* Supervised Exercise Group, *EORTC QLQ C-30* Quality of Life Questionnaire by the European Organization for Research and Treatment of Cancer, *MFI-20* Multidimensional Fatigue Inventory with 20 questions, *FAQ* Fatigue Assessment Questionnaire

**Table 3:** Studies with BCS results pre–post-intervention and percentage differences within groups (both intervention and control group)

Author-year	Type of exercise	Pre-value	Post-value	%Diff IG	%Diff CG
CRF					
Campbell et al. 2018	A	23.9 ± 7.0 ml/kg/min	27.3 ml/kg/min	+ 14.2	+ 1.1
Courneya et al. 2003	A	18.6 ± 3.9 ml/kg/min	21.3 ± 3.7 ml/kg/min	+ 14.5	− 3.2
Murtezani et al. 2014	A	799.6 ± 81.0 m	875.1 ± 86.7 m	+ 9.4	+ 1.1
Nikander et al. 2007	A	17.9 ± 1.5 min	17.6 ± 1.3 min	+ 1.7	+ 3.4
Northey et al. 2019 –HIIT	A	18.5 ± 3.9 ml/kg/min	22.0 ± 3.5 ml/kg/min	+ 18.9	− 2.9
Northey et al. 2019 –MOD	A	21.8 ± 3.4 ml/kg/min	23.1 ± 4.3 ml/kg/min	+ 6.0	/
Scott et al. 2020 -LET	A	21.5 ± 4.4 ml/kg/min	22.2 ± 4.6 ml/kg/min	+ 3.3	/
Scott et al. 2020 –NLET	A	22.2 ± 4.3 ml/kg/min	23.1 ± 4.8 ml/kg/min	+ 4.1	/
Dieli-Conwright et al. 2018	COMB	23.3 ± 6.1 ml/kg/min	35.1 ± 8.0 ml/kg/min	+ 50.6	− 15.0
Nikander et al. 2012	COMB	17.7 ± 2.0 min	16.9 ± 1.9 min	+ 4.5	+ 2.8
**Total**	**A**			** + 9.0**	**−** **0.1**
	**COMB**			** + 27.6**	**− 6.1**
ST					
Nikander et al. 2007	A	1246.0 ± 177.0 N	1305.0 ± 177.0 N	+ 4.7	+ 0.7
Hagstrom et al. 2016	RT	117.9 ± 41.6 kg	158.0 ± 45.6 kg	+ 33.9	+ 3.1
Dieli-Conwright et al. 2018-Leg Extension	COMB	45.4 ± 10.6 kg	75.7 ± 10.8 kg	+ 66.7	− 4.5
Dieli-Conwright et al. 2018-Leg Flexion	COMB	39.5 ± 9.6 kg	63.6 ± 11.2 kg	+ 61.0	− 2.7
Nikander et al. 2012	COMB	136.0 ± 23.0 kg	136.0 ± 23.0 kg	0	+ 1.5
**Total**	**A**			** + 4.7**	** + 0.7**
	**RT**			** + 33.9**	** + 3.1**
	**COMB**			** + 42.6**	− **1.9**
F					
Campbell et al. 2018	A	71.4 ± 21.1	76.1	− 6.6	− 0.7
Courneya et al. 2003	A	17.6 ± 11.5	8.3 ± 7.9	− 52.8	− 18.5
Saarto et al. 2012	A	40.5 ± 8.3	42.9	− 5.9	− 5.9
Scott et al. 2020 -LET	A	36.7 ± 11.9	39.5 ± 12.2	− 7.6	/
Scott et al. 2020 –NLET	A	42.8 ± 8.9	44.8 ± 9.0	− 4.7	/
Hagstrom et al. 2016	RT	39.1 ± 10.0	45.7 ± 7.6	− 16.9	− 4.0
Schmidt et al. 2012	RT	49.0 ± 23.7	26.0 ± 23	− 46.9	/
Stan et al. 2016	RT	13.6 ± 18.5	6.2	− 54.4	/
Dieli-Conwright et al. 2018	COMB	7.1 ± 2.0	2.9 ± 1.5	− 59.2	+ 6.9
Kiecolt-Glaser et al. 2014	Yoga	14.3 ± 19.6	6.2	− 56.6	− 40.5
** Total**	**A**			− **15.5**	− **8.4**
	**RT**			− **39.4**	− **4.0**
	**COMB**			− **59.2**	** + 6.9**
	**Yoga**			− **56.6**	− **40.5**
HRQoL					
Courneya et al. 2003 -FACT-G	A	85.5 ± 12.4	91.3 ± 11.0	+ 6.8	+ 0.6
Courneya et al. 2003 -FACT-B	A	110.5 ± 19.0	119.6 ± 16.9	+ 8.2	+ 0.3
Courneya et al. 2003 -TOI	A	70.8 ± 13.7	77.0 ± 12.0	+ 8.8	− 0.1
Murtezani et al. 2014 -FACT-G	A	77.4 ± 9.0	86.5 ± 7.3	+ 11.8	− 0.6
Murtezani et al. 2014 -FACT-B	A	99.8 ± 11.4	113.2 ± 9.7	+ 13.4	− 0.8
Saarto et al. 2012	A	69.8 ± 17.8	74.0	+ 6.0	+ 8.0
Scott et al. 2020 -LET- FACT-G	A	85.3 ± 13.6	87.1 ± 16.8	+ 2.1	/
Scott et al. 2020 -NLET-FACT-G	A	90.3 ± 11.6	93.8 ± 11.3	+ 2.9	/
Scott et al. 2020 -LET-FACT-B	A	104.8 ± 17.2	107.8 ± 20.7	+ 3.9	/
Scott et al. 2020—NLET-FACT-B	A	111.6 ± 14.1	116.7 ± 14.0	+ 4.6	/
Hagstrom et al. 2016	RT	89.1 ± 11.7	96.0 ± 8.7	+ 7.7	+ 1.8
Schmidt et al. 2012	RT	59.0 ± 16.6	76.0 ± 12.9	+ 28.8	/
Stan et al. 2016 -FACT-G	RT	83.3 ± 12.5	86.4	+ 3.7	/
Stan et al. 2016 -FACT-B	RT	110.9 ± 15.8	116.4	+ 5.0	/
Stan et al. 2016 –TOI	RT	68.9 ± 12.4	73.8	+ 7.1	/
Dieli-Conwright et al. 2018 -FACT-G	COMB	77.2 ± 9.0	88.3 ± 9.9	+ 14.4	− 0.4
Dieli-Conwright et al. 2018 -FACT-B	COMB	98.3 ± 14.1	113.0 ± 13.0	+ 15.0	− 2.4
Dieli-Conwright et al. 2018 -SF-36	COMB	66.1 ± 9.3	72.7 ± 10.5	+ 10.0	− 3.2
Odynets et al. 2019	Pilates	84.7 ± 2.6	117.0 ± 2.6	+ 44.5	/
Odynets et al. 2019	Yoga	82.5 ± 1.9	119.2 ± 3.1	+ 38.1	/
**Total**	**A**			** + 6.8**	** + 1.2**
	**RT**			** + 10.5**	** + 1.8**
	**COMB**			** + 13.1**	− **2.0**
	**Yoga**			** + 41.3**	**/**

Bold indicates percentage difference mean values for specific exercise intervention typology

+ increase*, − decrease*, * to be noted that regarding CRF, *ST and QoL* improved outcomes are represented by increases, while F symptoms represent improvements through decrease. *%Diff* percentage differences within group, *CRF* cardiorespiratory fitness, *ST* strength, *F* fatigue, *HRQoL* Health-Related Quality of Life, *A* aerobic training, *RT* resistance training, *COMB* combined aerobic and resistance training, *HIIT* high-intensity interval training, *MOD* Moderate intensity continuous aerobic exercise, *LET* linear intensity exercise training, *NLET* nonlinear intensity exercise training, *FACT-G/B* Functional Assessment of Cancer Treatment—General/Breast, *TOI* Trial outcome index score, *SF-36 GHI* Short Form Health Survey with 36 items

#### Aerobic

Of the four studies proposing A interventions, two studies implemented home-based walking/jogging activities [42, 43] while the other two used different aerobic training equipment [39, 41]. CRF [38, 39, 43], ST [39, 42], F [39, 41] and HRQoL [39, 41, 43], showed a mean difference of + 2.1%, + 9.7%, + 17.1% and + 4%, respectively (Table 2).

#### Resistance training

Six studies carried out RT interventions, [37, 38, 40–42, 44]. Three studies administered multiple exercises (averaging 2–3 sets, 8–12 repetitions at 50–80%1RM) [40, 41, 44] while the remaining applied just one exercise (4 sets, 4 repetitions at 85–90% 1RM) [37, 38] or unsupervised RT using resistance bands and tubing (2 sets, 8–10 reps for each exercise) [42]. CRF [38, 42], ST [37, 42], F [37, 40, 41, 44] and HRQoL [37, 40, 41, 44] showed a mean difference of + 6.4%, + 21.9%, + 5.9% and + 12.1%, respectively (Table 2).

#### Combined interventions

Only the study of Courneya et al. administered a COMB [39]. The study reported changes of − 13.1%, + 9.9%, + 14.3% and − 7.3% for CRF, ST, F and HRQoL, respectively (Table 2).

### Survivors

Table 3 summarizes results regarding the included studies with BCS and a specific training effect summary for each variable as well as the comparisons with CGs percentage difference. There were 14 included studies in BCS for a total sample of 1279 participants. Mean duration and frequency of interventions were 22 (range 10–48) weeks and 3 (range 1–4) sessions/week. Mean exercise intensity was 69% *V*O_2_max/Heart rate reserve and 60% 1RM, for A and RT interventions, respectively. There were seven studies that proposed A interventions [45, 46, 50, 51, 53, 55, 57], three studies that applied RT interventions [48, 56, 58], and two studies with COMB interventions [47, 52]. Furthermore, two studies with Yoga and Pilates protocols were included [49, 54].

#### Aerobic

In A interventions, four studies utilized different aerobic training equipment (treadmill, stationary bicycle and stair-climbing machine, cycle ergometer) [46, 50, 53, 57]. The remaining two studies included aerobic step, rope-jumping and skate-jumping exercises with additional walking or cycling [51, 55]. CRF [45, 46, 50, 51, 53, 57], ST [51], F [45, 46, 55, 57] and HRQoL [46, 50, 55, 57] showed a mean difference of + 9%, + 4.7%, − 15.5% and + 6.8%, respectively (Table 3).

#### Resistance training

Three studies implemented RT interventions. Two studies implemented both machine-based exercise and free-weight exercise (3sets, 8–10 repetitions, 6 different exercises per session) [48] or only workout machines (1 set, 20 repetitions, > 50% h1RM) [56]. The remaining study implemented home-based RT (administered via DVD) using resistance bands (8–10 repetitions, 5 upper and 5 lower-body exercises with additional core muscle engagement) [58]. None of the RT interventions assessed CRF. Only Hagstrom et al. provided ST measurements and showed improvements of + 33.9% [48]. All of the included studies [48, 56, 58] assessed F and HRQoL and showed a mean decrease of − 39.4% for F and a mean increase of + 10.5% for HRQoL (Table 3).

#### Combined interventions

Two studies implemented COMB interventions [47, 52]. The first study [47] administered two A sessions (treadmill running, rowing or cycling, 65–80% Maximal heart rate, 30–50 min) and one RT session/week (circuit training approach, 10–15 reps, 80% and 60% 1RM intensity for lower and upper-body exercises, respectively). The second study administered aerobic steps, rope-jumping, and skate-jumping along with additional walking or cycling (A) and dumbbell exercises for upper extremities (RT) [52]. Both studies assessed CRF and ST [47, 52]. They showed a + 27.6% mean improvement for CRF and a + 42.6% mean increase for ST. Only Dieli-Conwright et al. assessed F, and showed a − 59.2% mean reduction [47]. HRQoL showed a + 13.1% mean improvement (Table 3).

#### Pilates and yoga

Pilates and/or Yoga interventions studies were also screened but the majority was excluded from this review due to the frequently added meditation phase. For this reason, only two studies without meditation were included [49, 54]. The study of Kiecolt-Glaser et al. showed a reduction in F symptoms (56.6% and 40.5% in both IG and CG, respectively) after a 12-week (2 sessions/week) Yoga intervention [49]. The study of Odynets et al. showed improvements in HRQoL in response to one year (3 sessions/week) of Pilates or Yoga (+ 44.5% and + 38.1% in the Pilates and Yoga group, respectively) [54] (Table 3).

## Discussion

The purpose of this systematic review was to understand the isolated effects of exercise interventions on CRF, ST, F and HRQoL among BCP and BCS.

The main results show that exercise interventions among BCP, which were all in adjuvant therapy, were able to attenuate deteriorations in fitness and the symptom exacerbation displayed in the CGs. Interestingly, after RT protocols, there were minor increments of F symptoms (+ 5.95%) which were observed with a greater extent in all other protocols, and improvement of all other indices (by + 6.4%, + 21.9% and + 12.1%, for CRF, ST and HRQoL, respectively). However, we are not able to clearly understand the role of COMB protocols in BCP since only one study with COMB intervention was included.

Results regarding BCS exhibited improvements in indices of physical fitness following exercise interventions, while no changes were observed in the CGs. COMB and RT interventions for BCS showed encouraging data, with reductions in F and improvements in CRF, ST and HRQoL. Interestingly, RT interventions yielded higher percentage improvements in HRQoL and ST and also a more substantial reduction in F than the observed changes for A interventions. Positive effects were also observed for Pilates and Yoga interventions despite more studies are needed for corroboration.

### Cardiorespiratory fitness

CRF can be a useful indicator to understand how much side effects and sedentary choices contribute and are leading to health impairment [59]. From the included studies in this review, we observed overall positive results for both BCP and BCS.

Only the results from Courneya et al. demonstrated a reduction in CRF in BCP patients during adjuvant chemotherapy, yet they were able to demonstrate that a higher aerobic exercise dose was more effective than COMB and standard A interventions to avoid CRF decline [39]. Similarly, nonsignificant improvements were found in BCP after low to moderate intensity A by Segal et al. [43]. However, during exercise prescriptions it’s important to consider, that different outcomes can be found regarding CRF when different chemotherapy administrations occur [39, 60]. Therefore, exercise specialists should take into account different chemotherapy regimens considering that they could lead to different training outcomes [39].

Collectively, our results are consistent with a review from Maginador et al., in which moderate A interventions showed no effects on CRF, while a significant improvement was observed following high intensity A interventions for BCP during chemotherapy [59].

For BCS, A and COMB interventions also resulted in positive effects on CRF. A deviation from this observation occurred in Nikander et al.’s study, which showed a higher improvement in the CG than in the IG following an A intervention, although this was likely due to the recovery process after adjuvant therapy [51]. Still, high-intensity interval training interventions seem to be more effective than moderate A training [53], indicating that low-intensity A training may not be sufficient to obtain demonstrable changes among BCS.

### Strength

The ability to conduct activities of daily living could be limited by a lack of strength that, prior to diagnosis of the illness and the start of therapies, could be easily undertaken. While reductions in CRF decrease general activity, ST loss reduces functionality, which requires more external help and reduces the patient’s independence, leading to a decrease in HRQoL [61]. For these reasons, assessing and improving ST is crucial for designing effective training interventions in clinical trials and in practice [62, 63].

ST appeared to improve consistently in BCP, independently from the type of prescribed activity. Is important to note the ST improvement observed by Cešeiko et al. in the 1RM strength test (+ 19.1%) following 12 weeks (2 sessions/week) of maximal strength training (MST) performed on a horizontal dynamic leg press (intensity 85–90%1RM) [37]. This study demonstrated that MSTs are feasible, safe, and effective among BCP (when performed in supervised and individualized circumstances) [37]. Interestingly, even higher percentage improvements were found by Schwartz et al. (+ 22.8–24.7%), which was likely due to the longer intervention period (24 weeks) and higher weekly frequency (4 sessions/week) [42].

In general, there was a lack of studies assessing ST following both A and RT interventions among BCS. RT and COMB interventions demonstrated higher mean percentage improvements (+ 33.9% and + 42.6% in RT and COMB, respectively) than A interventions (+ 4.7%), but more evidence is needed to corroborate these findings.

Overall, our results showed that exercise interventions at least maintain fitness levels for BCP and improve it for BCS, with promising substantial responses from RT interventions. These results are in alignment with the review from Montaño-Rojas et al. and the review from Strasser et al. in which ST improvements were found in either BCS and BCP [64, 65].

### Fatigue

F is one of the most frequent symptoms and side effects of cancer therapy [9, 10]. It is also one of the first factors that could initiate the vicious cycle established between symptoms, side effects, and the resultant sedentary lifestyle that contributes to a patient’s CRF and ST loss [8]. Thus, several studies have focused on the effects of exercise on cancer-related fatigue. According to a review by Kessels et al., which assessed the effects of exercise on F among cancer survivors, exercise can both directly affect F since it counteracts deconditioning (restoring CRF and ST) and indirectly affect F by mitigating F-associated conditions (e.g. insomnia, pain, anxiety and depression) [66]. The authors of this review suggest A activities (focusing on patients adherence) to manage F, because it showed greater effects when compared with other and low-adherence interventions [66]. These results are in contrast with our review, in which RT and COMB interventions presented overall higher percentage reductions in F when compared to A to manage fatigue in BCP and BCS. To be noted that the review by Kessels et al. did not independently evaluate RT as stand-alone exercise interventions [66].

When examining RT, two studies implemented similar interventions and used the Fatigue Assessment Questionnaire among BCP during adjuvant radiotherapy [44] or chemotherapy [40]. The promising outcomes in these studies are mainly due to the physical dimension of fatigue (while no significant effects were found among affective and cognitive dimensions) [40, 44]. These results are confirmed by the review of van Vulpen et al. among BCP during adjuvant therapy [67]. Thus, in addition to exercise other complementary interventions could be necessary during adjuvant therapy to also improve cognitive and affective fatigue dimensions in BCP.

All studies conducted in BCS observed reductions in F-symptomology. This is probably due to the absence of current therapy side effects that may exacerbate fatigue. RT interventions appeared to further improve F levels when compared to A interventions. Consistently, a recent review of systematic reviews by Jiang et al. on cancer-related fatigue management through exercise in BCS, showed that both A and RT are helpful [68].

### Health-related quality of life

Improving physical fitness (in both CRF and ST) and symptom management, especially F, could lead to an improved HRQoL [61]. Implementing a healthy and active lifestyle could increase patients’ social interactions, leading to an improved psychological condition. Questionnaires implemented in cancer exercise trials usually include questions regarding tiredness, sleep/resting necessities, ability to work or perform simple to complex exercise tasks [69–71]. Thus, assessing HRQoL is very important because it allows caregivers to understand whether or not the physiological improvements (e.g. on CRF and ST) are related to a better everyday life.

In our work, all of the studies conducted in BCP showed improvements in HRQoL, except for Courneya et al. which found a reduction in HRQoL in every group, which was most likely due to the chemotherapy side effects [39].

All the included studies concerning BCS participants presented HRQoL improvements in IGs while decreases were found in CGs. The only exception was the study of Saarto et al. in which HRQoL improvements in both IG and CG were probably caused by CG motivation (“high RoB” judgement) [55]. Also the meta-analysis by Zhu et al. showed similar results to ours regarding the effects of exercise on HRQoL in BCS. The authors showed additional beneficial effects of exercise on depression, anxiety, body composition, muscle strength and physiological markers. However, a broad variety of exercise interventions were included making it hard to identify the effects of isolated exercise interventions [72].

Overall, our results indicate that exercise interventions have a positive effect on HRQoL on both BCP and BCS. Two other Cochrane reviews analyzed the effect of exercise among BCP during and after adjuvant therapy, respectively [73, 74]. The first review found that exercise during adjuvant therapy yielded small, if any improvements, in HRQoL and improved cancer site-specific HRQoL [73]. The second review found small-to-moderate improvements on general HRQoL [74]. Another review from Gebruers et al. assessed the effects of exercise on BCP during treatment [24]. Similar to the findings of this review, the results of Gebruers et al. highlight that RT and COMB protocols are able to further manage F and improve fitness compared to A interventions while HRQoL was the least influenced outcome after exercise [24].

Our review presented some limitations. We aimed to understand the isolated effect of exercise interventions on BCP and BCS. However, it is not possible to control patients in their everyday life and avoid deviations from the intended interventions that may have biased the results. Fortunately, the majority of the interventions involved supervised programs. Additionally, the included studies usually involved patients without exercise contraindications or disabilities which may, in some cases, have predisposed the analysis to include patients with a higher physical fitness level.

Another limitation of this study was the impossibility to include studies that assessed the effects of exercise among BCP during neoadjuvant therapy, or among patients with BC related long-term side effects (e.g. lymphedema and aromatase inhibitor arthralgia). It is probable that BCP necessitate specific multidimensional approaches either when they are administered with neoadjuvant therapy or when long-term side effects occur [75, 76].

There were also no eligible studies with patients with metastatic BC included. However, thanks to the results showed by Singh et al.’s review, we also know that exercise is safe and effective for stage II + local, regional and distant BCP patients [26].

Despite the abovementioned limitations, our work showed important results similar to the review of McNeely et al., who found exercise as an effective treatment in BCP and BCS to improve CRF, physical functioning, fatigue and quality of life, notwithstanding a small number of trials included [20]. In our review, we expanded upon previous works by including results pertaining to either breast cancer patients and survivors for each administered intervention, obtaining a reasonable number of included studies. However, well-designed studies with large samples are required to better define the exercise guidelines for this specific population.

In conclusion, a structured exercise program seems to be a useful strategy for preventing the exacerbation of cancer symptoms and the deterioration of physical fitness and health-related quality of life among breast cancer patients during adjuvant therapy. Exercise can also reduce fatigue symptoms and improve cardiorespiratory fitness, strength, and health-related quality of life in breast cancer survivors. Our results recommend resistance training and combined aerobic-resistance training interventions for positive changes to the evaluated outcomes. However, exercise prescriptions should be delivered and initially supervised by trained exercise specialists. Knowledge regarding breast cancer patients is essential to design optimal and individualized exercise protocols that allow a gradual and safe progression of components of physical fitness.

## Supplementary Information

Below is the link to the electronic supplementary material.

Supplementary file1 (PDF 95 KB)

## Abbreviations

1RM: 1-Repetition maximum
A: Aerobic intervention
BC: Breast cancer
BCP: Breast cancer patients
BCS: Breast cancer survivors
CG: Control Group
COMB: Combined A + RT intervention
CRF: Cardiorespiratory fitness
F: Fatigue
HRQoL: Health-related quality of life
IG: Intervention group
MST: Maximal strength training
RCT: Randomized controlled trial
RoB: Risk of bias
RT: Resistance training intervention
ST: Strength
*V*O_2_max: Maximal oxygen consumption

## References

[1] WHO World Health Organization. Global Health Estimates 2020: deaths by cause, age, sex, by country and by region, 2000–2019. 2020; Available from: https://www.who.int/data/gho/data/themes/mortality-and-global-health-estimates/ghe-leading-causes-of-death. Cited 15 Mar 2020.

[2] Sung H, Ferlay J, Siegel RL, Laversanne M, Soerjomataram I, Jemal A (2021). Global cancer statistics 2020: GLOBOCAN estimates of incidence and mortality worldwide for 36 cancers in 185 countries. CA Cancer J Clin.

[3] Akram M, Iqbal M, Daniyal M, Khan AU (2017). Awareness and current knowledge of breast cancer. Biol Res.

[4] Parker PA, Youssef A, Walker S, Basen-Engquist K, Cohen L, Gritz ER (2007). Short-term and long-term psychosocial adjustment and quality of life in women undergoing different surgical procedures for breast cancer. Ann Surg Oncol.

[5] Shapiro CL, Recht A (2001). Side effects of adjuvant treatment of breast cancer. N Engl J Med.

[6] Yu AF, Jones LW (2016). Breast cancer treatment-associated cardiovascular toxicity and effects of exercise countermeasures. Cardiooncology.

[7] Neil-Sztramko SE, Kirkham AA, Hung SH, Niksirat N, Nishikawa K, Campbell KL (2014). Aerobic capacity and upper limb strength are reduced in women diagnosed with breast cancer: a systematic review. J Physiother.

[8] Dimeo FC (2001). Effects of exercise on cancer-related fatigue. Cancer.

[9] Smets EM, Garssen B, Schuster-Uitterhoeve AL, de Haes JC (1993). Fatigue in cancer patients. Br J Cancer.

[10] Mock V (2001). Fatigue management: evidence and guidelines for practice. Cancer.

[11] Shien T, Iwata H (2020). Adjuvant and neoadjuvant therapy for breast cancer. Jpn J Clin Oncol.

[12] Siegel RL, Miller KD, Jemal A (2020). Cancer statistics, 2020. CA Cancer J Clin.

[13] Malvezzi M, Carioli G, Bertuccio P, Boffetta P, Levi F, La Vecchia C (2019). European cancer mortality predictions for the year 2019 with focus on breast cancer. Ann Oncol.

[14] Rietman JS, Dijkstra PU, Debreczeni R, Geertzen JH, Robinson DP, De Vries J (2004). Impairments, disabilities and health related quality of life after treatment for breast cancer: a follow-up study 2.7 years after surgery. Disabil Rehabil.

[15] Elme A, Utriainen M, Kellokumpu-Lehtinen P, Palva T, Luoto R, Nikander R (2013). Obesity and physical inactivity are related to impaired physical health of breast cancer survivors. Anticancer Res.

[16] Kokkonen K, Saarto T, Mäkinen T, Pohjola L, Kautio H, Järvenpää S (2017). The functional capacity and quality of life of women with advanced breast cancer. Breast Cancer.

[17] Mehta LS, Watson KE, Barac A, Beckie TM, Bittner V, Cruz-Flores S (2018). Cardiovascular disease and breast cancer: where these entities intersect: a scientific statement from the American Heart Association. Circulation.

[18] Gernaat SAM, Ho PJ, Rijnberg N, Emaus MJ, Baak LM, Hartman M (2017). Risk of death from cardiovascular disease following breast cancer: a systematic review. Breast Cancer Res Treat.

[19] Palesh O, Scheiber C, Kesler S, Mustian K, Koopman C, Schapira L (2018). Management of side effects during and post-treatment in breast cancer survivors. Breast J.

[20] McNeely ML, Campbell KL, Rowe BH, Klassen TP, Mackey JR, Courneya KS (2006). Effects of exercise on breast cancer patients and survivors: a systematic review and meta-analysis. CMAJ.

[21] Moore SC, Lee IM, Weiderpass E, Campbell PT, Sampson JN, Kitahara CM (2016). Association of leisure-time physical activity with risk of 26 types of cancer in 1.44 million adults. JAMA Intern Med.

[22] Lynch BM, Neilson HK, Friedenreich CM (2011). Physical activity and breast cancer prevention. Recent Results Cancer Res.

[23] McTiernan A, Friedenreich CM, Katzmarzyk PT, Powell KE, Macko R, Buchner D (2019). Physical activity in cancer prevention and survival: a systematic review. Med Sci Sports Exerc.

[24] Gebruers N, Camberlin M, Theunissen F, Tjalma W, Verbelen H, Van Soom T (2019). The effect of training interventions on physical performance, quality of life, and fatigue in patients receiving breast cancer treatment: a systematic review. Support Care Cancer.

[25] Lee J, Lee MG (2020). Effects of exercise interventions on breast cancer patients during adjuvant therapy: a systematic review and meta-analysis of randomized controlled trials. Cancer Nurs.

[26] Singh B, Spence RR, Steele ML, Sandler CX, Peake JM, Hayes SC (2018). A systematic review and meta-analysis of the safety, feasibility, and effect of exercise in women with stage II+ breast cancer. Arch Phys Med Rehabil.

[27] Spei ME, Samoli E, Bravi F, La Vecchia C, Bamia C, Benetou V (2019). Physical activity in breast cancer survivors: a systematic review and meta-analysis on overall and breast cancer survival. Breast.

[28] Campbell KL, Winters-Stone KM, Wiskemann J, May AM, Schwartz AL, Courneya KS (2019). Exercise guidelines for cancer survivors: consensus statement from international multidisciplinary roundtable. Med Sci Sports Exerc.

[29] Hayes SC, Newton RU, Spence RR, Galvão DA (2019). The Exercise and Sports Science Australia position statement: exercise medicine in cancer management. J Sci Med Sport.

[30] Moher D, Liberati A, Tetzlaff J, Altman DG, Group P (2009). Preferred reporting items for systematic reviews and meta-analyses: the PRISMA statement. PLoS Med.

[31] Burstein HJ, Lacchetti C, Anderson H, Buchholz TA, Davidson NE, Gelmon KA (2019). Adjuvant Endocrine Therapy for Women With Hormone Receptor-Positive Breast Cancer: ASCO Clinical Practice Guideline Focused Update. J Clin Oncol.

[32] Thomas E, Bellafiore M, Gentile A, Paoli A, Palma A, Bianco A (2021). Cardiovascular responses to muscle stretching: a systematic review and meta-analysis. Int J Sports Med.

[33] Lin X, Zhang X, Guo J, Roberts CK, McKenzie S, Wu WC (2015). Effects of exercise training on cardiorespiratory fitness and biomarkers of cardiometabolic health: a systematic review and meta-analysis of randomized controlled trials. J Am Heart Assoc.

[34] Wilder RP, Greene JA, Winters KL, Long WB, Gubler K, Edlich RF (2006). Physical fitness assessment: an update. J Long Term Eff Med Implants.

[35] Haraldstad K, Wahl A, Andenæs R, Andersen JR, Andersen MH, Beisland E (2019). A systematic review of quality of life research in medicine and health sciences. Qual Life Res.

[36] Akl E, Altman D, Aluko P, Askie L, Beaton D, Berlin J et al. Cochrane handbook for systematic reviews of interventions 2019.

[37] Cešeiko R, Eglitis J, Srebnijs A, Timofejevs M, Purmalis E, Erts R (2019). The impact of maximal strength training on quality of life among women with breast cancer undergoing treatment. Exp Oncol.

[38] Cešeiko R, Thomsen SN, Tomsone S, Eglītis J, Vētra A, Srebnijs A (2020). Heavy resistance training in breast cancer patients undergoing adjuvant therapy. Med Sci Sports Exerc.

[39] Courneya KS, McKenzie DC, Mackey JR, Gelmon K, Friedenreich CM, Yasui Y (2013). Effects of exercise dose and type during breast cancer chemotherapy: multicenter randomized trial. J Natl Cancer Inst.

[40] Schmidt ME, Wiskemann J, Armbrust P, Schneeweiss A, Ulrich CM, Steindorf K (2015). Effects of resistance exercise on fatigue and quality of life in breast cancer patients undergoing adjuvant chemotherapy: a randomized controlled trial. Int J Cancer.

[41] Schmidt T, Weisser B, Dürkop J, Jonat W, Van Mackelenbergh M, Röcken C (2015). Comparing endurance and resistance training with standard care during chemotherapy for patients with primary breast cancer. Anticancer Res.

[42] Schwartz AL, Winters-Stone K, Gallucci B (2007). Exercise effects on bone mineral density in women with breast cancer receiving adjuvant chemotherapy. Oncol Nurs Forum.

[43] Segal R, Evans W, Johnson D, Smith J, Colletta S, Gayton J (2001). Structured exercise improves physical functioning in women with stages I and II breast cancer: results of a randomized controlled trial. J Clin Oncol.

[44] Steindorf K, Schmidt ME, Klassen O, Ulrich CM, Oelmann J, Habermann N (2014). Randomized, controlled trial of resistance training in breast cancer patients receiving adjuvant radiotherapy: results on cancer-related fatigue and quality of life. Ann Oncol.

[45] Campbell KL, Kam JWY, Neil-Sztramko SE, Liu Ambrose T, Handy TC, Lim HJ (2018). Effect of aerobic exercise on cancer-associated cognitive impairment: a proof-of-concept RCT. Psychooncology.

[46] Courneya KS, Mackey JR, Bell GJ, Jones LW, Field CJ, Fairey AS (2003). Randomized controlled trial of exercise training in postmenopausal breast cancer survivors: cardiopulmonary and quality of life outcomes. J Clin Oncol.

[47] Dieli-Conwright CM, Courneya KS, Demark-Wahnefried W, Sami N, Lee K, Sweeney FC (2018). Aerobic and resistance exercise improves physical fitness, bone health, and quality of life in overweight and obese breast cancer survivors: a randomized controlled trial. Breast Cancer Res.

[48] Hagstrom AD, Marshall PW, Lonsdale C, Cheema BS, Fiatarone Singh MA, Green S (2016). Resistance training improves fatigue and quality of life in previously sedentary breast cancer survivors: a randomised controlled trial. Eur J Cancer Care (Engl).

[49] Kiecolt-Glaser JK, Bennett JM, Andridge R, Peng J, Shapiro CL, Malarkey WB (2014). Yoga's impact on inflammation, mood, and fatigue in breast cancer survivors: a randomized controlled trial. J Clin Oncol.

[50] Murtezani A, Ibraimi Z, Bakalli A, Krasniqi S, Disha ED, Kurtishi I (2014). The effect of aerobic exercise on quality of life among breast cancer survivors: a randomized controlled trial. J Cancer Res Ther.

[51] Nikander R, Sievänen H, Ojala K, Oivanen T, Kellokumpu-Lehtinen PL, Saarto T (2007). Effect of a vigorous aerobic regimen on physical performance in breast cancer patients—a randomized controlled pilot trial. Acta Oncol.

[52] Nikander R, Sievänen H, Ojala K, Kellokumpu-Lehtinen PL, Palva T, Blomqvist C (2012). Effect of exercise on bone structural traits, physical performance and body composition in breast cancer patients—a 12-month RCT. J Musculoskelet Neuronal Interact.

[53] Northey JM, Pumpa KL, Quinlan C, Ikin A, Toohey K, Smee DJ (2019). Cognition in breast cancer survivors: a pilot study of interval and continuous exercise. J Sci Med Sport.

[54] Odynets T, Briskin Y, Todorova V (2019). Effects of different exercise interventions on quality of life in breast cancer patients: a randomized controlled trial. Integr Cancer Ther.

[55] Saarto T, Penttinen HM, Sievänen H, Kellokumpu-Lehtinen PL, Hakamies-Blomqvist L, Nikander R (2012). Effectiveness of a 12-month exercise program on physical performance and quality of life of breast cancer survivors. Anticancer Res.

[56] Schmidt T, Weisser B, Jonat W, Baumann FT, Mundhenke C (2012). Gentle strength training in rehabilitation of breast cancer patients compared to conventional therapy. Anticancer Res.

[57] Scott JM, Thomas SM, Peppercorn JM, Herndon JE, Douglas PS, Khouri MG (2020). Effects of exercise therapy dosing schedule on impaired cardiorespiratory fitness in patients with primary breast cancer: a randomized controlled trial. Circulation.

[58] Stan DL, Croghan KA, Croghan IT, Jenkins SM, Sutherland SJ, Cheville AL (2016). Randomized pilot trial of yoga versus strengthening exercises in breast cancer survivors with cancer-related fatigue. Support Care Cancer.

[59] Maginador G, Lixandrão ME, Bortolozo HI, Vechin FC, Sarian LO, Derchain S (2020). Aerobic exercise-induced changes in cardiorespiratory fitness in breast cancer patients receiving chemotherapy: a systematic review and meta-analysis. Cancers (Basel)..

[60] Courneya KS, Segal RJ, Mackey JR, Gelmon K, Reid RD, Friedenreich CM (2007). Effects of aerobic and resistance exercise in breast cancer patients receiving adjuvant chemotherapy: a multicenter randomized controlled trial. J Clin Oncol.

[61] Kärki A, Simonen R, Mälkiä E, Selfe J (2005). Impairments, activity limitations and participation restrictions 6 and 12 months after breast cancer operation. J Rehabil Med.

[62] Campbell KL, Pusic AL, Zucker DS, McNeely ML, Binkley JM, Cheville AL (2012). A prospective model of care for breast cancer rehabilitation: function. Cancer.

[63] Cantarero-Villanueva I, Fernández-Lao C, Díaz-Rodríguez L, Fernández-de-Las-Peñas C, Ruiz JR, Arroyo-Morales M (2012). The handgrip strength test as a measure of function in breast cancer survivors: relationship to cancer-related symptoms and physical and physiologic parameters. Am J Phys Med Rehabil.

[64] Montaño-Rojas LS, Romero-Pérez EM, Medina-Pérez C, Reguera-García MM, de Paz JA (2020). Resistance training in breast cancer survivors: a systematic review of exercise programs. Int J Environ Res Public Health.

[65] Strasser B, Steindorf K, Wiskemann J, Ulrich CM (2013). Impact of resistance training in cancer survivors: a meta-analysis. Med Sci Sports Exerc.

[66] Kessels E, Husson O, van der Feltz-Cornelis CM (2018). The effect of exercise on cancer-related fatigue in cancer survivors: a systematic review and meta-analysis. Neuropsychiatr Dis Treat.

[67] van Vulpen JK, Peeters PH, Velthuis MJ, van der Wall E, May AM (2016). Effects of physical exercise during adjuvant breast cancer treatment on physical and psychosocial dimensions of cancer-related fatigue: a meta-analysis. Maturitas.

[68] Jiang M, Ma Y, Yun B, Wang Q, Huang C, Han L (2020). Exercise for fatigue in breast cancer patients: an umbrella review of systematic reviews. Int J Nurs Sci.

[69] Aaronson NK, Ahmedzai S, Bergman B, Bullinger M, Cull A, Duez NJ (1993). The European Organization for Research and Treatment of Cancer QLQ-C30: a quality-of-life instrument for use in international clinical trials in oncology. J Natl Cancer Inst.

[70] Ware JE, Sherbourne CD (1992). The MOS 36-item short-form health survey (SF-36). I. Conceptual framework and item selection. Med Care.

[71] Cella DF, Tulsky DS, Gray G, Sarafian B, Linn E, Bonomi A (1993). The Functional Assessment of Cancer Therapy scale: development and validation of the general measure. J Clin Oncol.

[72] Zhu G, Zhang X, Wang Y, Xiong H, Zhao Y, Sun F (2016). Effects of exercise intervention in breast cancer survivors: a meta-analysis of 33 randomized controlled trails. Onco Targets Ther.

[73] Furmaniak AC, Menig M, Markes MH (2016). Exercise for women receiving adjuvant therapy for breast cancer. Cochrane Database Syst Rev.

[74] Lahart IM, Metsios GS, Nevill AM, Carmichael AR (2018). Physical activity for women with breast cancer after adjuvant therapy. Cochrane Database Syst Rev.

[75] Ezzo J, Manheimer E, McNeely ML, Howell DM, Weiss R, Johansson KI (2015). Manual lymphatic drainage for lymphedema following breast cancer treatment. Cochrane Database Syst Rev.

[76] Roberts K, Rickett K, Greer R, Woodward N (2017). Management of aromatase inhibitor induced musculoskeletal symptoms in postmenopausal early breast cancer: a systematic review and meta-analysis. Crit Rev Oncol Hematol.

