# Supplementary material for: Impact of exercise interventions on physical fitness in breast cancer patients and survivors: a systematic review
Source: Breast Cancer. 2022 Mar 12;29(3):402–18. doi: 10.1007/s12282-022-01347-z (PMC9021138; doi:10.1007/s12282-022-01347-z)
Supplement: Supplementary file 1 — Supplementary file1 (PDF 95 KB) [file 12282_2022_1347_MOESM1_ESM.pdf]

Impact of exercise interventions on physical fitness in breast cancer patients and survivors: A Systematic Review

Authors

Salvatore Ficarra[1]* MSc, Ewan Thomas[1] PhD, Antonino Bianco[1] PhD, Ambra Gentile[1] MSc, Petra Thaller[2] MSc, Fulvio Grassadonio[3] MSc, Sofia Papakonstantinou[4] MSc, Thorsten Schulz[5] PhD, Nils Olson[5] MSc, Alexandra Martin[6] MSc, Christian Wagner[7] MSc, Anna Nordstrom[8-9] MD, PhD, Hande Hofmann[5] PhD

*Corresponding author:

Salvatore Ficarra, MSc

Orcid-ID: 0000-0001-9975-6542

salvatore.ficarra03@unipa.it

Sport and Exercise Sciences Research Unit

Department of Psychology, Educational Science and Human Movement

University of Palermo

Via Giovanni Pascoli 6 - 90144, Palermo

Tel. 0039 3406264547

Palermo, Italy

Supplementary file:

1. Search strategy for one database

2. Levels of evidence and grades of recommendations

1. Search strategy for one database

Population

Woman diagnosed with breast cancer in treatment or survivors [‘breast cancer’, ‘breast neoplasm’]

Interventions

Exercise or physical activity protocols [‘exercise’, ‘physical activity’]

Comparators

Usual care, wait-list control, other exercise interventions

Outcomes

Cardiorespiratory fitness, Strength, Fatigue, Quality of life

Study design

Randomized Controlled Trials

Limitations

Peer-review papers, published between January 2000 and November 2020

Filters

Publication Date, Clinical Trial, Randomized Controlled Trial (Medline); Publication Date, Article, English (Scopus)

Keywords

1. Breast Cancer
2. Breast Neoplasm
3. Exercise
4. Physical Activity

Medline

#	Searches	Results
1	1 AND 3	787
2	1 AND 4	855
3	2 AND 3	742
4	2 AND 4	805

The search was conducted up to the 22th of November 2020, the same approach was adopted for all the screened database.

2. Levels of evidence and grades of recommendations

Author	Level of Evidence	Grades of Recommendation
Campbell et al	2b	C
Cešeiko et al 2019	1b	A
Cešeiko et al 2020	2b	B
Courneya et al 2003	1b	A
Courneya et al 2013	1b	A
Dieli-Conwright et al	1b	A
Hagstrom et al	2b	C
Kiecolt-Glaser et al	1b	A
Murtezani et al	2b	B
Nikander et al 2007	2b	C
Nikander et al 2012	2b	B
Northey et al	2b	C
Odynets et al	2b	B
Saarto et al	2b	C
Schmidt M. E. 2015	1b	A
Schmidt 2012	2b	C
Schmidt T. 2015	2b	C
Schwartz et al	1b	A
Scott et al	1b	A
Segal et al	1b	A
Stan et al	2b	C
Steindorf et al	1b	A

Level of Evidence 1A= Systematic review (with homogeneity) of RCTs; 1B=Individual RCT (with narrow confidence intervals); 1C=All or none study; 2A=Systematic review (with homogeneity) of cohort studies; 2B= Individual Cohort study (including low quality RCT, e.g. <80% follow-up); 2C="Outcomes" research; Ecological studies; 3A= Systematic review (with homogeneity) of case-control studies; 3B=Individual Case-control study; 4= Case series (and poor quality cohort and case-control study; 5=Expert opinion without explicit critical appraisal or based on physiology bench research or "first principles" *From the Centre for Evidence-Based Medicine, http://www.cebm.net. Grades of Recommendation A= Level 1, Strong recommendation; B=Levels 2, 3 or 4, Recommendation; C=Levels 2, 3 or 4, Option; D=Level 5, Option. From American Society of Plastic Surgeons Evidence-based clinical practice guidelines. Available at: https://www.plasticsurgery.org/documents/medical-professionals/health-policy/evidence-practice/ASPS-Scale-for-Grading-Recommendations.pdf. Accessed February 12, 2021.
